# Stability of Nitrogen-Doped Activated Carbon as an Electrocatalyst for the Oxygen Reduction Reaction in Various Storage Media

**DOI:** 10.3390/molecules29153611

**Published:** 2024-07-30

**Authors:** Tao Zhang, Songlin Zuo

**Affiliations:** 1 International Innovation Center for Forest Chemicals and Materials, College of Chemical Engineering, Nanjing Forestry University, Nanjing 210037, China; zt20170709@njfu.edu.cn; 2 Jiangsu Co-Innovation Center of Efficient Processing and Utilization of Forest Resources, Nanjing Forestry University, Nanjing 210037, China

**Keywords:** nitrogen-doped carbon materials, storage, stability, catalytic activity, pyridinic N, graphitic N

## Abstract

Besides outstanding catalytic performance, the stability of nitrogen-doped carbon materials during storage is equally crucial for practical applications. Therefore, we conducted the first investigation into the stability of highly nitrogen-doped activated carbon (AC-NC-T) obtained by modifying activated carbon with CO_2_/NH_3_ in different storage media (air, vacuum and N_2_). The results of the catalysis of the oxygen reduction reaction and the activation of peroxymonosulfate for degrading bisphenol A by AC-NC-T show that the catalytic activity of AC-NC-T stored in air decays most prominently, while the performance attenuated only marginally when stored in vacuum and N_2_. The results from N_2_ adsorption isotherms, Raman spectroscopy, elemental and X-ray photoelectron spectroscopy indicate that the decline in catalytic activity is due to the presence of oxygen in the environment, causing a decrease in absolute contents of pyridinic N (N-6) and graphitic nitrogen (N-Q). After being stored in an air atmosphere for 28 days, the absolute contents of N-6 and N-Q in AC-NC-950 decreased by 19.3% and 12.1%, respectively. However, when stored in a vacuum or N_2_, the reduction in both was less than 7%. This study demonstrates that reducing oxygen concentration during storage is crucial for preserving high catalytic activity of nitrogen-containing carbon materials.

## 1. Introduction

Substitutional doping of heteroatoms (for example, N, S, P, B) is a feasible strategy for improving the electronic and chemical properties of carbon materials such as graphene [1,2], carbon nanotubes (CNTs) [3,4], carbon aerogels [5,6], mesoporous carbon [7] and activated carbon [3,8]. Nitrogen is the most widely studied heteroatom because it has atomic radii similar to carbon atoms, and thus allows for it to be more easily incorporated into carbon materials without significant crystal lattice misalignment [9,10]. Furthermore, the introduction of nitrogen into the carbon matrix disrupts its electronic and spin properties, thus, breaking the chemical inertness of the carbon-based materials [2,10,11], which enhances the surface wettability, modifies the electronic structure and reduces energy barriers for ion adsorption and desorption, greatly expanding the carbon materials applications in the fields of adsorption and separation [12,13], advanced oxidation process (AOPs) [8,14,15], energy storage and conversion [9,16] and catalysis [17,18]. In 2016, Bao’s group [19] reported a novel layered nitrogen-doped porous carbon with a record-high Henry’s law CO_2_/N_2_ selectivity of 124:1 at 298 K and 1 bar, along with high CO_2_ adsorption capacity (4.50 mmol/g). The strategy proposed in this study has garnered widespread attention and catalyzed the further advancement of carbon-based adsorbents. In the field of water pollution remediation, Duan et al. [8] found that nitrogen-doped carbon material, as a metal-free catalyst, can activate peroxymonosulfate by a non-radical pathway. Dai’s group [20] discovered that vertically aligned nitrogen-doped CNTs (NCNTs), as a non-metallic electrode, exhibit superior electrocatalytic activity, long-term stability and tolerance compared to platinum. This discovery inspired considerable investigations to develop high-performance and cost-effective carbon-doped materials for the oxygen reduction reaction (ORR) electrocatalyst. There exist numerous techniques for the fabrication of nitrogen-doped carbon materials, beyond the traditional thermal treatment [21], such as chemical vapor deposition [22], template method [23] and self-assembly method [24,25].

Although N-doped carbon materials have attracted extensive attention from the scientific and industrial community due to their excellent performance, the stability of such functional materials during use and storage is equally critical. However, current research primarily focuses on the stability of the nitrogen-doped materials during use, with little attention being paid to their stability during storage [7,26,27,28]. Herein, we investigated for the first time the stability of a series of NH_3_/CO_2_-modified activated carbons with high nitrogen content stored in different media (air, vacuum and N_2_). The catalytic performance towards the ORR and ability to activate peroxymonosulfate (PMS) to degrade bisphenol A (BPA) by N-doped activated carbon was used as the probe reaction to evaluate the evolution in their catalytic activity during storage. The results of the ORR experiment and the BPA degradation experiment indicate that the attenuation of catalytic performance in nitrogen-doped activated carbon stored in air is the most pronounced, whereas it is minimal when stored in vacuum and in a N_2_ atmosphere, particularly in the N_2_ environment. This study demonstrates that the storage atmosphere is quite important to keep the high catalytic performance of the N-containing carbon materials and helps to extend the lifespan of such materials.

## 2. Results and Discussion

### 2.1. Study on the Stability of N-Doped Activated Carbons in Air Storage

#### 2.1.1. Catalytic ORR Performance of Samples

**Figure 1:**
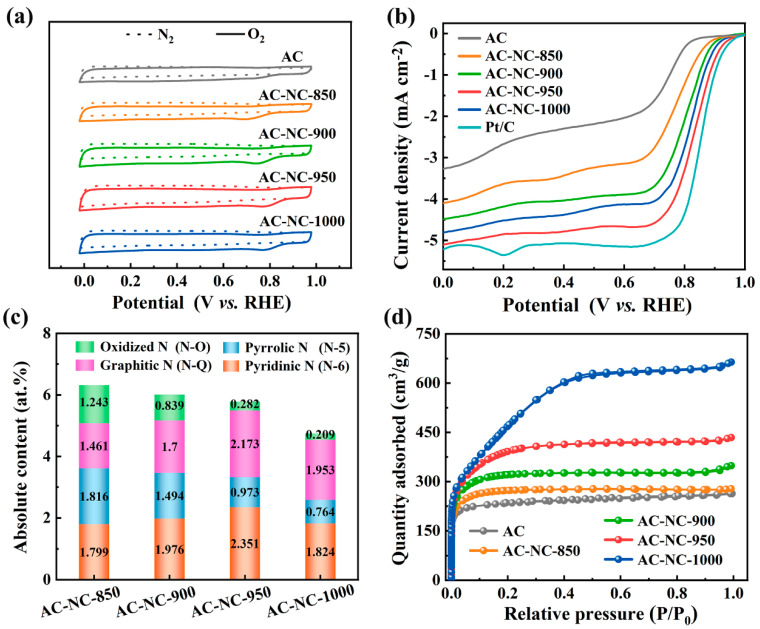
(**a**) CV curves of AC and AC–NC–T in N_2_ and O_2_–saturated 0.1 M KOH solution at a scan rate of 50 mV·s^−1^. (**b**) LSV curves of AC and AC-NC-T in O_2_–saturated 0.1 M KOH solution at a scan rate of 10 mV·s^−1^ and 1600 rpm. (**c**) Absolute nitrogen content of AC–NC–T. (**d**) N_2_ adsorption–desorption isotherms of AC and AC–NC–T.

The catalytic activity towards the ORR of AC, AC-NC-850, AC-NC-900, AC-950 and AC-NC-1000 was investigated by measuring the CV curves in an N_2_ or O_2_-saturated 0.1 M KOH solution. As depicted in Figure 1a, in a N_2_-saturated 0.1 M KOH solution, the CV curves of all these catalysts do not show an evident peak; however, in the O_2_-saturated electrolyte solution, they all show a typical reduction peak, indicating their capability for catalyzing oxygen reduction. The ORR peaks at 0.674 V, 0.722 V, 0.759 V, 0.781 V and 0.798 V vs. RHE could be found for AC, AC-NC-850, AC-NC-900, AC-NC-950 and AC-NC-1000, respectively. Notably, the cathodic peak potential of the AC-NC-950 was more positive compared to that of the other samples, while the more positive peak potential represents a smaller overpotential for the ORR and better catalytic performance. Therefore, according to the comparison of the positions of the oxygen reduction peaks, the order of the ORR catalytic performance of the samples should be AC-NC-950 > AC-NC-1000 > AC-NC-900 > AC-NC-850 > AC. This trend can be further confirmed by the LSV tests performed on a rotating disk electrode in O_2_-saturated 0.1 M KOH at a rotating speed of 1600 rpm and scan rate of 10 mV s^−1^. As shown in Figure 1b, the onset (E_onset_) and half-wave (E_1/2_) potential, and the limiting current density (J_L_) of the AC-NC-950 are 0.946 V vs. RHE, 0.827 V vs. RHE and −5.122 mA·cm^−2^, respectively, surpassing those of the other three samples significantly and approaching that of Pt/C (Table 1).

Relative to other samples, the improvement of the AC-NC-950 on the ORR activity is ascribed to its highest absolute content of N-6 and N-Q and the second well-developed pore structure (Figure 1c,d). It has been reported that both N-6 and N-Q could act as the active sites for the ORR, which could lower the overpotential and increase the current density [16,29,30,31]. Figure 1c and Figure S1 illustrate the substantial presence of N-6 and N-Q in AC-NC-950, with absolute contents reaching 2.351 at. % and 2.173 at. %, respectively, and thus provides an ample array of active sites that are essential to facilitate the ORR. Since the ORR only occurs at the triphase interface between the active site, electrode and electrolyte [32,33], the catalyst needs not only sufficient active sites but also a well-developed pore structure to fully expose these active sites and optimize the delivery of reactants and electrolytes [34,35]. Based on Figure 1d and Figure S2, it is evident that the specific surface area and mesoporous volume of AC-NC-950 rank second only to those of AC-NC-1000 among all the samples. These attributes are advantageous for the exposure of active sites and facilitation of electrolyte and oxygen mass transfer within the pores, thereby enhancing the efficiency of the oxygen reduction reaction [21,36,37]. Therefore, it is the high N-6 and N-Q absolute content and well-developed pore structure of the AC-NC-950 that work together to give it better electrochemical activity than other samples.

#### 2.1.2. Storage in an Air Environment

**Figure 2:**
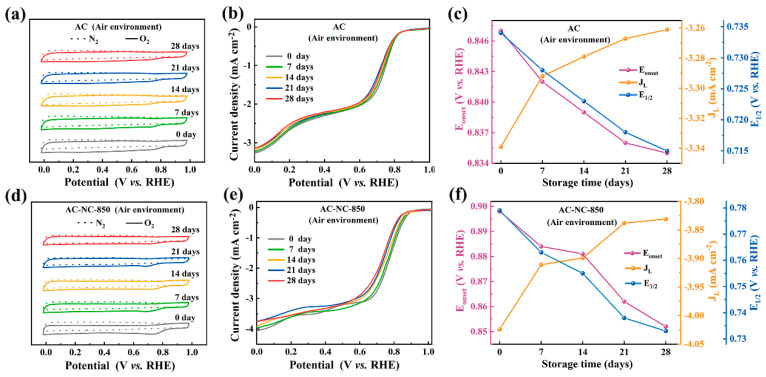

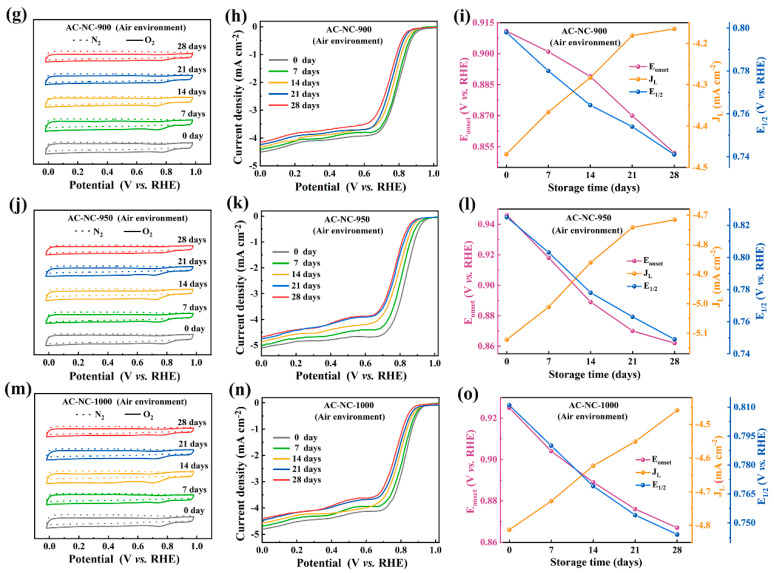
(**a**,**d**,**g**,**j**,**m**) Changes in the CV curves of AC and AC–NC–T with different storage times in air in N_2_ and O_2_–saturated 0.1 M KOH solution (scan rate of 50 mV s^−1^). (**b**,**e**,**h**,**k**,**n**) LSV curves of AC and AC–NC–T stored in air for different times in O_2_-saturated 0.1 M KOH (scan rate 10 mV s^−1^, rotation rate 1600 rpm). (**c**,**f**,**i**,**l**,**o**) E_onset_, E_1/2_ and J_L_ of the corresponding LSV curves.

The N-doped activated carbons were stored in an air atmosphere for 7, 14, 21 and 28 days at room temperature. Their CV and LSV curves are shown in Figure 2. As shown in Figure 2, the intensity and electric potential of the redox peak in the CV curves of AC-NC-T gradually decreased with the extension of storage time. This indicates that air exposure leads to a decline in the ORR electrocatalytic ability. Figure 2 shows that the LSV curves of carbon samples changed obviously due to the storage in air. The variation in the E_onset_, E_1/2_, and J_L_ of the activated carbons with the storage time are presented in Figure 2. It is evident that the ORR electrocatalytic activity of these N-doped activated carbons deteriorate with the extension of the storage time in air. Even in the initial 7 days, their ORR electrocatalytic activity obviously decreased. After storage in air for 28 days, the E_onset_ of AC, AC-NC-850, AC-NC-900, AC-NC-950 and AC-NC-1000 decreased by 1.42%, 5.12%, 6.48%, 8.88% and 6.27%, respectively, while the E_1/2_ decreased by 2.58%, 5.90%, 7.14%, 9.43% and 8.26%, respectively. The J_L_ also decreased by 2.34%, 4.80%, 6.78%, 7.93% and 7.37%, respectively. It is noted that the electrocatalytic activity of AC-NC-950 is the most obviously decreased. In contrast, the undoped carbon sample shows minimal changes. This implies that the nitrogen-containing groups in the activated carbons are responsible for the attenuation of the electrocatalytic activity caused by the storage in air.

### 2.2. Evolution of Surface Chemical Properties of N-Doped Activated Carbon Upon Exposure to Air

**Figure 3:**
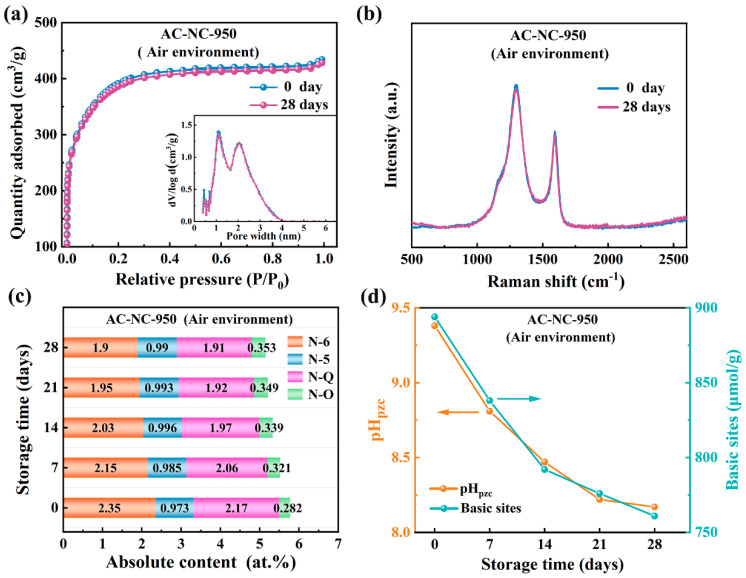
Evolution of pore structure (**a**) and Raman spectra (**b**) of AC–NC–950 after 28 days air storage. (**c**) The absolute nitrogen content of AC–NC–950 stored in a vacuum environment changes over times. (**d**) The alkaline properties of AC–NC–950 following storage in air for different times.

Figure 3 shows the changes in pore structure, microstructure, N-containing species content and the alkaline properties of the AC-NC-950 in the process of storage in air at room temperature and atmospheric pressure for 28 days. The N_2_ adsorption–desorption isotherms and Raman spectra demonstrated that the pore structure and microstructure of the AC-NC-950 had negligible changes after 28 days of storage (Figure 3a,b). Table S2 lists the elemental content of the AC-NC-950 being stored in air for the various days. The elemental analysis showed that the content of C in AC-NC-950 had negligible change, while the content of O was minimally increased and N was minimally decreased. The results from the XPS analysis confirmed that the change in the elements C, O, N on the surface of the samples had the same trend during the storage in air (Table S2). This indicated that the oxygen was chemically adsorbed in the samples. The deconvolution of the XPS N1s spectra revealed that the absolute content of the N-containing species was obviously changed (Figure 3c and Figure S3). Specifically, the content of N-6 and N-Q was gradually decreased but the species of oxidized N (N-O) was increased and pyrrolic N (N-5) was nearly unchanged during storage in air (Figure 3c). When the sample AC-NC-950 was stored in air for 28 days, the absolute contents of N-6 and N-Q were reduced by 19.3% and 12.1%, respectively. Therefore, the evolution of the nitrogen-containing groups demonstrated that oxygen in air are apt to be chemically adsorbed in N-6 and N-Q rather than N-5 forming oxidized N components N-O. This finding was also consistent with a previous report [9,38]. This is because the nitrogen atoms in N-6 and N-Q have higher electron density, and thus, higher activity than that in N-5 [35,39]. Additionally, it is noted that the content of N-6 had a slightly greater decrease than N-Q, which was possibly caused by the difference in the location of N-Q and N-6 in the carbon sample. The species of N-Q are basically located in the interior of the graphitic domains, while N-6 is mainly in the edge of graphitic domains. The N-6 and N-Q atoms located at the edge of the carbon network typically exhibit higher catalytic activity and possess a structure more suitable for oxygen adsorption [9]. When N-6 and N-Q are attacked by oxygen in the air, a transition state is formed, which is the critical stage of a chemical reaction. As the reaction proceeds, the bond structure between atoms changes, leading to the occurrence of oxidation reactions. During this process, the rearrangement of electrons and sharing between atoms leads to the formation of new species, accompanied by the release or absorption of energy. Ultimately, oxidized nitrogen is formed. Meantime, a decrease in the content of N-6 and N-Q lead to a decrease in the basicity and pH_pzc_. Figure 3d shows that the content of basic sites and pH_pzc_ of the AC-NC-950 were decreased from 894 μmol/g to 761 μmol/g and 9.38 to 8.17, respectively, when stored in air for 28 days. The theoretical study has demonstrated that the N-6 species in the carbon materials is characteristic of superior basicity as both a Lewis base and a Brønsted base [40]. Accordingly, the change in the basicity and pH_pzc_ confirmed the change in the N-containing species during storage in air.

This development provides a reasonable explanation for the fact that the storage in air impairs the electrocatalytic activity of the AC-NC-950. It was previously reported that the increased N-6 content and N-Q is beneficial for improving the E_onset_ and the J_L_ of the ORR, respectively. As a result, when stored in air for 28 days, the E_onset_ of the AC-NC-950 decreased from 0.946 V to 0.862 V and the J_L_ decreased from −5.122 mA cm^−2^ to −4.716 mA cm^−2^ (Figure 2l). The decrease in the E_onset_ is greater than the decrease in the J_L_, which is consistent with the changes in the absolute content of N-6 and N-Q. Moreover, we standardized the catalytic oxygen reduction performance of AC-NC-950 stored for varying durations in an air atmosphere, and the results are depicted in Figure S4. As shown in Figure S4, it is evident that the R^2^ values of the fitting curves for both specific potential and limiting current density exceed 0.93, indicating high reliability and stability of the experiment data. This observation suggests that catalytic sites which have not yet undergone passivation retain their catalytic activity during storage.

### 2.3. Stability of N-Doped Activated Carbon under Vacuum and N_2_ Environment

**Figure 4:**
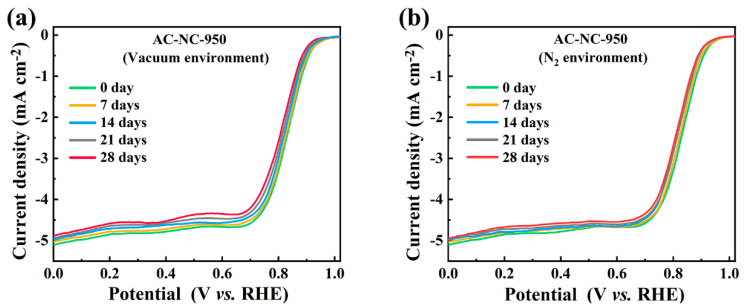
LSV curves of AC–NC–950 stored in vacuum atmosphere (**a**) and N_2_ atmosphere (**b**) for different time in O_2_–saturated 0.1 M KOH (scan rate 10 mV s^−1^, rotation rate 1600 rpm).

Figure 4a shows the LSV curves of AC-NC-950 after storage under vacuum within 28 days and the corresponding electrochemical parameters are listed in Table S3. The catalytic performance towards the ORR of AC-NC-950 was much more stable in the case of storage under vacuum than that in air. After storage under vacuum for 28 days, the E_onset_, E_1/2_ and J_L_ of AC-NC-950 were minimally changed with 2.87%, 3.14%, and 3.07%, respectively (Table S3). The elemental and XPS analysis showed that the C content and the absolute content of N-5 were minimally changed (Table S2 and Figure S5). The change in the N-6, N-Q and N-O content of AC-NC-950 were much less than in the case of storage in air. This slight change in the electrocatalytic performance and the N-containing species was caused by the trace of residual oxygen under vacuum with a vacuum degree of −0.95 bar, which is chemically adsorbed in the N-containing groups with a higher electron density. Correspondingly, the basic sites and pH_pzc_ of AC-NC-950 were slightly decreased during storing under vacuum (Figure S5f). Therefore, a high vacuum degree favors storing N-doped activated carbons under vacuum for retention of their high catalytic activity.

Figure 4b presents the LSV curves of AC-NC-950 stored in a N_2_ atmosphere for varying durations. After storage for 28 days, AC-NC-950-air-28 exhibited almost identical LSV curves to those of freshly prepared AC-NC-950. Meanwhile, the values of E_onset_, E_1/2_ and J_L_ hardly changed (Table S3). This was because the concentration of oxygen in the N_2_ atmosphere was significantly lower than in a vacuum or air environment. The XPS analysis indicated that the sample stored in a N_2_ atmosphere for 28 days exhibited significantly smaller changes in N and O content compared to samples stored in vacuum or air (Table S2). The variations in the contents of both elements did not exceed 4%, and the absolute contents of N-6 and N-Q were lower than 7%, thereby leading to only trivial changes in the alkaline site content and pH_pzc_ of the sample (Figure S6f). The above results demonstrated that AC-NC-950, with the highest catalytic activity, exhibited high stability in the electrocatalytic ORR performance and structure in a N_2_ atmosphere over a long period of storage (28 days).

### 2.4. BPA Degradation Experiment

**Figure 5:**
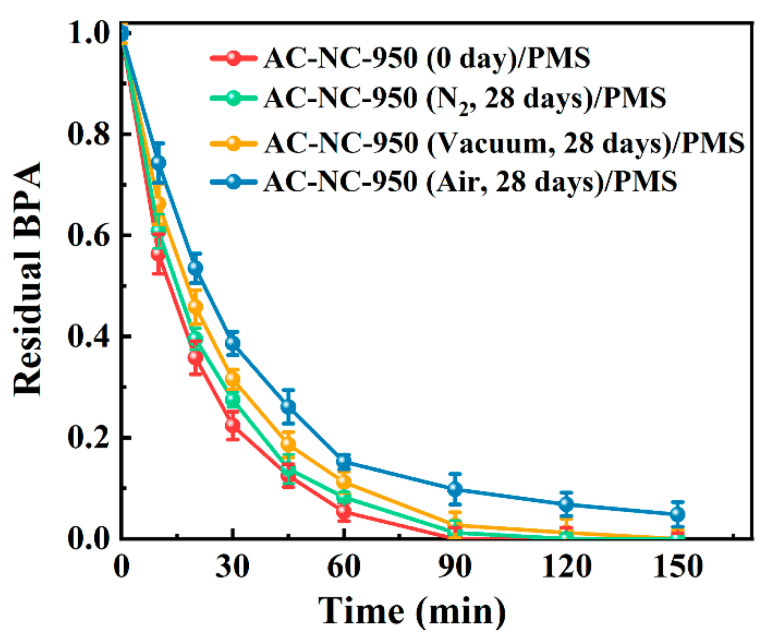
Evolution of BPA during catalyzed PMS activation with sample AC–NC–950 stored in different media for 28 days, in comparison with newly prepared A AC–NC–950. Conditions: [BPA] = 50 mg/L, [catalyst] = 0.15 g/L, [PMS] = 5 mM, initial pH = 5, T = 25 °C.

Numerous studies have demonstrated the outstanding catalytic capabilities of N-doped carbon materials in activating peroxides to degrade organic pollutants in aquatic environments [14,41,42]. Accordingly, we examined the catalytic activity of AC-NC-950 in activating PMS to degrade BPA after storage in air, under vacuum and under a N_2_ atmosphere for 28 days to further verify the impact of storage on the activity and stability of N-doped carbon materials. The removal efficiency of BPA using AC-NC-950 as the catalyst of PMS is shown in Figure 5. It is evident that the newly prepared AC-NC-950 can catalytically activate PMS to completely remove BPA in the aqueous solution within 90 min. However, when AC-NC-950 was stored in air for 28 days, only 91.4% of BPA was removed even within 150 min. Also, it is seen that the BPA removal efficiency was a little decreased when AC-NC-950 was stored under vacuum or a N_2_ atmosphere. This indicated that the storage of AC-NC-950 in air resulted into the most significant deterioration of the catalytic activity for activation to degrade BPA, with a little decrease for the storage under vacuum and a N_2_ atmosphere. These results are consistent with the situation of the electrocatalytic performance in the process of storing under different atmosphere.

## 3. Materials and Methods

### 3.1. Materials and Chemicals

The commercial coconut shell activated carbon was supplied by Ruineng Carbon Material Technology Co. Ltd. (Huzhou, Zhejiang, China). Potassium hydroxide (KOH, ≥97%), sodium hydroxide (NaOH, ≥97%), hydrochloric acid (HCl, 36–38%) and sodium chloride (NaCl, ≥99%) were of analytical grade and obtained from Nanjing Chemical Reagent Co. Ltd. (Nanjing, Jiangsu, China). Phenolphthalein (98%), bisphenol A (BPA, ≥98%), peroxymonosulfate (KHSO_5_, ≥42%) and methanol (HPLC grade) were purchased from Macklin Biochemical Co. Ltd. (Shanghai, China). Ultrapure water (18.25 MΩ·cm) was provided by Zhituo IQ-3 purifying water system (Zhituo instrument Co. Ltd. (Nanjing, Jiangsu, China)).

### 3.2. Preparation and Storage Methods for Nitrogen-Doped Activated Carbon

#### 3.2.1. Preparation of Nitrogen-Doped Activated Carbon

This study aimed to evaluate the impact of nitrogen-doped catalysts on the ORR activity. To obtain a uniform dispersion of catalyst ink for electrochemical testing, the catalyst needs to be pulverized or ball-milled and screened through a 400-mesh sieve. However, the pulverizing or ball-milling process may induce mechanical chemical reactions [43,44], which can change the physical and chemical properties of the carbon material and affect its electrochemical performance [45,46,47]. Therefore, to eliminate this influence, in this work, only the portion that passed through the 400-mesh sieve was used as the initial carbon material for subsequent nitrogen doping modification.

The screened activated carbon needs to be de-ashed before use. Powdered activated carbon is first added to 0.1 M HCl solution and subsequently rinsed thoroughly with boiled ultrapure water until the electrical conductivity of the washings was less than 10 μS/cm. The ash content of the obtained activated carbon was reduced from 4.2% to 0.19%. The de-ashed activated carbon is designated as AC.

The nitrogen doping of AC was conducted in a tube furnace. Briefly, 1.5 g of AC (<400-mesh) was heated at a rate of 5 °C/min to a given temperature (850 °C, 900 °C, 950 °C, 1000 °C) in a mixture of N_2_ (99.999%, 40 mL/min) and CO_2_ (99.99%, 40 mL/min). When the set temperature was reached, the atmosphere was switched to the mixture of NH_3_ (99.999%, 80 mL/min) and CO_2_ (99.99%, 40 mL/min), and held at the given temperature for 90 min. The obtained sample was named AC-NC-T, where T represents the final treatment temperature.

#### 3.2.2. Storage Conditions of Carbon Samples

To investigate the effect of the storage media on the physicochemical stability of the samples, the freshly prepared samples were designated as the samples of 0 day and were promptly stored in the air, vacuum and N_2_ environment, respectively. The samples were placed flat on Petri dishes and then placed in three dryers, each corresponding to a different atmosphere. To reduce container humidity in the dryers, 2000 g of silica gel was placed in each dryer, which were equipped with a digital thermo-hygrometer (971LS3, Fluke, Everett, WA, USA) to monitor temperature and humidity changes. Throughout the experiment, all dryers were kept in a room with air conditioning and a dehumidifier (DH-8138C, Changzhou, China), maintaining the room temperature at 25 °C and relative humidity between 33% and 53%. During the experiment, samples are required to be taken out for characterization and testing separately on the 7th, 14th, 21st and 28th days.

When the storage medium inside the dryer was air, the relative humidity fluctuated within a range of 33–48% during the 28-day experimental period. When the storage medium inside the dryer was a vacuum, the vacuum degree inside the dryer was set to −0.96 bar, and the relative humidity within the vacuum dryer fluctuated between 33% and 45% within 28 days. In the preliminary experiment, the vacuum degree in the vacuum dryer decreased by <8% within 28 days, verifying the dryer’s good sealing performance. When the sample storage atmosphere was high-purity N_2_, a micro-negative pressure environment was provided for the dryer and the vacuum degree was set to −0.1 bar. The vacuum pressure gauge data intuitively reflects pressure changes inside the dryer, allowing for assessment of its sealing performance. During the 28-day test period, the relative humidity within the dryer remained within the range of 32% to 41%.

### 3.3. Electrode Preparation and Electrochemical Measurements

The carbon stored in three different environments (air, vacuum and N_2_) was directly used as catalysts to prepare electrodes. Their catalytic ORR activity was measured with an electrochemical workstation from Zennium (Zahner Electrochemistry, Kronach, Bavaria, Germany), with a conventional three-electrode system and 0.1 M KOH solution at room temperature. Pt wire and Ag/AgCl (saturated KCl) served as the counter and reference electrodes, respectively, while a glassy carbon electrode (Pine Research Instrumentation, Grove, PA, USA) with a diameter of 5 mm and a geometric area of 0.196 cm^2^ was employed as the working electrode.

Prior to the electrochemical experiments, 4 mg of AC-NC-T was dispersed in 0.95 mL ethanol/water solution (volume ratio of 7:3), then sonicated for 1 h. Then, 50 μL of 0.5% Nafion solution was added and sonicated for 1 h to obtain a uniform catalyst ink. Then, 10 µL of the ink was dropped onto a well-cleaned glassy carbon electrode and the electrode was transferred to a vacuum drying oven and dried at 25 °C for 12 h. The final catalyst loading was 0.2 mg/cm^2^. For comparison, the same amount of commercial Pt/C with a Pt loading of 20 wt. % (Premetek, Cherry Hill, NJ, USA) was loaded on the glass carbon electrode by the above method. During the ORR performance evaluation, high-purity O_2_ (99.999%) or N_2_ (99.999%) was continuously injected into the electrolyte solution to ensure gas saturation.

Cyclic voltammograms (CV) were conducted in N_2_/O_2_-saturated 0.1 M KOH solution at a scanning rate of 50 mV s^−1^. Linear sweep voltammograms (LSV) were recorded in an O_2_-saturated 0.1 M KOH solution at a rotational speed of 1600 r/min with a scan rate of 10 mV s^−1^. The CV and LSV for ORR were scanned in a negative direction. At the same time, background subtraction was performed under high-purity N_2_ saturation conditions. The potential was converted from Ag/AgCl (saturated KCl) to a reversible hydrogen electrode (RHE) by Equation (1).

E (V) _RHE_ = E (V)_Ag/AgCl_ + 0.197 + 0.059 × pH (1)

### 3.4. BPA Degradation Experiments

Degradation experiments were conducted at 25 °C in beakers containing 200 mL BPA solution with a stirring speed of 200 rpm. In a typical procedure, reactions were initiated by simultaneously adding catalysts (0.15 g/L) and PMS (5 mM) into the solution (50 mg/L). The initial pH of BPA solution was approximately 5.6. At pre-determined intervals, 1 mL of the solution was withdrawn and 0.5 mL methanol was added for quenching free radicals and residual oxidants. Thereafter, the solution was immediately filtered with 0.22 μm hydrophilic filter. Each experiment was repeated three times. The concentration of BPA was quantitatively analyzed by high performance liquid chromatography and the chromatographic conditions are described in detail in Supporting Information Text S1.

### 3.5. Characterization

The N_2_ adsorption/desorption isotherms of the carbon catalysts were measured at 77 K with a surface area and porosity analyzer (Autosorb IQ2, Quantachrome, Boston, MA, USA). Specific surface area (S_BET_) and micropore volume (V_mic_) of the carbons were obtained using the Brunauer–Emmett–Teller equation and Dubinin–Radushkevic equation, respectively. The total pore volume (V_t_) was obtained at P/P_0_ = 0.99. The mesopore volume (V_mes_) was calculated by subtracting V_mic_ from V_t_. The pore size distribution of the samples were analyzed using the method of quenched solid density functional theory. Raman spectra was taken on a Raman spectrometer (Thermo Fisher Scientific, Waltham, MA, USA) with 488 nm laser irradiation. The elemental composition of the carbons was analyzed for carbon, hydrogen, nitrogen, and oxygen using an elemental analyzer (Vario EL Cube, Elementar, Hanau, Germany). The surface composition and chemical state of the samples was determined by X-ray photoelectron spectroscopy (XPS, ESCALAB 250Xi, Thermo Fisher Scientific, USA) using an Al Kα monochromatic source.

The content of the acidic and basic surface active sites of the carbons were determined following the procedure described elsewhere [48]. The specific determination process is comprehensively outlined in Supporting Information Text S2. The point of zero charge (pH_pzc_) of the carbons was determined by the method proposed by Vega and Valdés [49]. The specific process of determination is described in detail in Supporting Information Text S3.

## 4. Conclusions

In this study, the effect of the storage atmosphere (air, vacuum, and N_2_) on the catalytic performance of the N-doped activated carbon was studied by examining its electrocatalytic ORR activity and its ability to activate PMS to degrade BPA. The results showed that the catalytic activity experiences a pronounced decline when N-doped activated carbon is exposed to air. Conversely, exposure to vacuum and N_2_ atmospheres results in only marginal degradation. This deterioration was caused by the oxygen present around the nitrogen-containing carbon materials during storage. The higher the oxygen concentration, the more pronounced the passivation phenomenon. Essentially, the elemental and XPS analysis demonstrated that the oxygen present in the storage environment during storage lead to a decrease in the absolute content of the N-6 and N-Q, thereby imparting the catalytic activity. Therefore, this study demonstrated that the nitrogen-containing carbon materials are required to be stored in an oxygen-free circumstance for retention of a high activity.

## Figures and Tables

**Table 1:** Oxygen reduction activity parameters of carbon samples.

Samples	E_onset_ (V vs. RHE)	E_1/2_ (V vs. RHE)	J_L_ (mA cm^−2^)
AC	0.847	0.734	−3.339
AC–NC–850	0.898	0.779	−4.024
AC–NC–900	0.911	0.798	−4.468
AC–NC–950	0.946	0.827	−5.122
AC–NC–1000	0.925	0.811	−4.813
Pt/C	0.961	0.857	−5.332

## Data Availability

Data will be made available upon request.

## Supplementary Materials

The following are available online at https://www.mdpi.com/article/10.3390/molecules29153611/s1, Figure S1: (a) XPS survey spectrum and (b) high resolution N 1s of N-doped activated carbons. Figure S2: Pore size distributions of the activated carbons. Figure S3: High-resolution N 1s of XPS spectra of AC-NC-950 stored under air atmosphere for varying durations. Figure S4: The evolution of the specific electrocatalytic activity of AC-NC-950 stored under air atmosphere. Figure S5: (a–d) High-resolution XPS spectra of N 1s of the AC-NC-950 stored under vacuum atmosphere for varying durations. (e) The absolute content of each nitrogen-containing component in AC-NC-950 changes over time when stored in an air atmosphere. (f) The alkaline properties of AC-NC-950 following storage in vacuum environment for varying durations. Figure S6: (a–d) High-resolution XPS spectra of N 1s of the AC-NC-950 stored under a N2 atmosphere for varying durations. (e) The absolute nitrogen content of AC-NC-950 stored in a N2 environment changes over times. (f) The alkaline properties of AC-NC-950 following storage in a N2 environment for varying durations. Table S1: Textural and surface chemistry of AC and AC-NC-T. Table S2a: The elemental content changes of AC-NC-950 after being stored in different media (air, vacuum, and N2) for different periods of time, as measured by elemental analysis. Table S2b: The elemental content changes of AC-NC-950 after being stored in different media (air, vacuum, and N2) for different periods of time, as measured by XPS. Table S3: The LSV curve parameters obtained from AC-NC-950 after being stored in a vacuum and a N2 atmosphere for various durations.

## References

[1] Samantaray S., Mohanty D., Satpathy S.K., Hung I.M. (2024). Exploring Recent Developments in Graphene-Based Cathode Materials for Fuel Cell Applications: A Comprehensive Overview. Molecules.

[2] Inagaki M., Toyoda M., Soneda Y., Morishita T. (2018). Nitrogen-doped carbon materials. Carbon.

[3] Byeon A., Yun W.C., Kim J.M., Lee J.W. (2023). Recent progress in heteroatom-doped carbon electrocatalysts for the two-electron oxygen reduction reaction. Chem. Eng. J..

[4] Hu C., Paul R., Dai Q., Dai L. (2021). Carbon-based metal-free electrocatalysts: From oxygen reduction to multifunctional electrocatalysis. Chem. Soc. Rev..

[5] Liu Y., Zhang X., Gu X., Wu N., Zhang R., Shen Y., Zheng B., Wu J., Zhang W., Li S. (2020). One-step turning leather wastes into heteroatom doped carbon aerogel for performance enhanced capacitive deionization. Microporous Mesoporous Mater..

[6] Yan Y., Wang H., Bi X., Zhao Y., Wu M. (2024). Efficient electrocatalytic reduction of CO_2_ to CO enhanced by the synergistic effect of N,P on carbon aerogel. Chem. Commun..

[7] Carrillo-Rodríguez J.C., Garay-Tapia A.M., Escobar-Morales B., Escorcia-García J., Ochoa-Lara M.T., Rodríguez-Varela F.J., Alonso-Lemus I.L. (2021). Insight into the performance and stability of N-doped Ordered Mesoporous Carbon Hollow Spheres for the ORR: Influence of the nitrogen species on their catalytic activity after ADT. Int. J. Hydrogen Energy.

[8] Duan X., Sun H., Wang S. (2018). Metal-Free Carbocatalysis in Advanced Oxidation Reactions. Acc. Chem. Res..

[9] Li J., Xia Z., Wang X., Feng C., Zhang Q., Chen X.A., Yang Y., Wang S., Jin H. (2024). Distinguished Roles of Nitrogen-Doped Sp^2^ and Sp^3^ Hybridized Carbon on Extraordinary Supercapacitance in Acidic Aqueous Electrolyte. Adv. Mater..

[10] Zhang P., Yang Y., Duan X., Liu Y., Wang S. (2021). Density Functional Theory Calculations for Insight into the Heterocatalyst Reactivity and Mechanism in Persulfate-Based Advanced Oxidation Reactions. ACS Catal..

[11] Ghosh S., Barg S., Jeong S.M., Ostrikov K. (2020). Heteroatom-Doped and Oxygen-Functionalized Nanocarbons for High-Performance Supercapacitors. Adv. Energy Mater..

[12] Liang W., Luo Z., Liu Z., Wei X., Cai W. (2023). Nitrogen-doped carbon particles with distinctive ethylene adsorption selectivity for efficient ethylene/acetylene separation. Chem. Eng. J..

[13] Zhang T., Zuo S. (2023). Drying enables multiple reuses of activated carbon without regeneration. Environ. Sci. Pollut. Res..

[14] Di L., Wang T., Lu Q., Lu J., Zhang Y., Zhou Y., Zhou Y. (2024). Efficient PMS activation toward degradation of bisphenol A by metal-free nitrogen-doped hollow carbon spheres. Sep. Purif. Technol..

[15] Yang L., Jiao Y., Xu X., Pan Y., Su C., Duan X., Sun H., Liu S., Wang S., Shao Z. (2022). Superstructures with Atomic-Level Arranged Perovskite and Oxide Layers for Advanced Oxidation with an Enhanced Non-Free Radical Pathway. ACS Sustain. Chem. Eng..

[16] Feng X., Bai Y., Liu M., Li Y., Yang H., Wang X., Wu C. (2021). Untangling the respective effects of heteroatom-doped carbon materials in batteries, supercapacitors and the ORR to design high performance materials. Energy Environ. Sci..

[17] Guo D., Shibuya R., Akiba C., Saji S., Kondo T., Nakamura J. (2016). Active sites of nitrogen-doped carbon materials for oxygen reduction reaction clarified using model catalysts. Science.

[18] Xu X., Pan Y., Zhong Y., Ran R., Shao Z. (2020). Ruddlesden–Popper perovskites in electrocatalysis. Mater. Horiz..

[19] To J.W.F., He J., Mei J., Haghpanah R., Chen Z., Kurosawa T., Chen S., Bae W.-G., Pan L., Tok J.B.H. (2016). Hierarchical N-Doped Carbon as CO_2_ Adsorbent with High CO_2_ Selectivity from Rationally Designed Polypyrrole Precursor. J. Am. Chem. Soc..

[20] Gong K., Du F., Xia Z., Durstock M., Dai L. (2009). Nitrogen-Doped Carbon Nanotube Arrays with High Electrocatalytic Activity for Oxygen Reduction. Science.

[21] Huang Q., Hu L., Chen X., Cai W., Wang L., Wang B. (2023). Metal–Organic Framework-Derived N-Doped Carbon with Controllable Mesopore Sizes for Low-Pt Fuel Cells. Adv. Funct. Mater..

[22] Dong H., Zhang L., Liao Y., Huang K., Lian C., Zhou X., Zhang Z., Kauppinen E.I., Wu Z.-S. (2023). Floating Catalyst Chemical Vapor Deposition Patterning Nitrogen-Doped Single-Walled Carbon Nanotubes for Shape Tailorable and Flexible Micro-Supercapacitors. Adv. Funct. Mater..

[23] Li J., Bao A. (2022). Self-sacrificing template synthesis of nitrogen-doped hierarchical porous carbons as an effective adsorbent for CO_2_ capture. J. Porous Mater..

[24] Sanchez-Ballester N.M., Rydzek G., Pakdel A., Oruganti A., Hasegawa K., Mitome M., Golberg D., Hill J.P., Abe H., Ariga K. (2016). Nanostructured polymeric yolk–shell capsules: A versatile tool for hierarchical nanocatalyst design. J. Mater. Chem. A.

[25] Li Y., Xie M., Zhang S., Zhao L., Kong L., Zhan J., Zhao R.-S. (2022). Porous 3D superstructure of nitrogen doped carbon decorated with ultrafine cobalt nanodots as peroxymonosulfate activator for the degradation of sulfonamides. Chem. Eng. J..

[26] Oh W.-D., Lisak G., Webster R.D., Liang Y.-N., Veksha A., Giannis A., Moo J.G.S., Lim J.-W., Lim T.-T. (2018). Insights into the thermolytic transformation of lignocellulosic biomass waste to redox-active carbocatalyst: Durability of surface active sites. Appl. Catal. B Environ..

[27] Wang S., Xia Y., Tan L., Wu S., Yu Y., Yu X., Guan Z., Chen H., Jiang F. (2023). Unraveling the instability of Nitrogen-Doped carbon during BPA treatment by peroxymonosulfate Activation: Effect of free radical grafting. Sep. Purif. Technol..

[28] Quílez-Bermejo J., Morallón E., Cazorla-Amorós D. (2022). On the deactivation of N-doped carbon materials active sites during oxygen reduction reaction. Carbon.

[29] Liu X., Dai L. (2016). Carbon-based metal-free catalysts. Nat. Rev. Mater..

[30] Choi C.H., Lim H.-K., Chung M.W., Park J.C., Shin H., Kim H., Woo S.I. (2014). Long-Range Electron Transfer over Graphene-Based Catalyst for High-Performing Oxygen Reduction Reactions: Importance of Size, N-doping, and Metallic Impurities. J. Am. Chem. Soc..

[31] Lv Q., Si W., He J., Sun L., Zhang C., Wang N., Yang Z., Li X., Wang X., Deng W. (2018). Selectively nitrogen-doped carbon materials as superior metal-free catalysts for oxygen reduction. Nat. Commun..

[32] Zhang Q., Zhou M., Ren G., Li Y., Li Y., Du X. (2020). Highly efficient electrosynthesis of hydrogen peroxide on a superhydrophobic three-phase interface by natural air diffusion. Nat. Commun..

[33] Zhang X., Xia Y., Xia C., Wang H. (2020). Insights into Practical-Scale Electrochemical H_2_O_2_ Synthesis. Trends Chem..

[34] Quílez-Bermejo J., Pérez-Rodríguez S., Torres D., Canevesi R., Morallón E., Cazorla-Amorós D., Celzard A., Fierro V. (2024). Nitrogen sites prevail over textural properties in N-doped carbons for the oxygen reduction reaction. J. Colloid Interface Sci..

[35] Encalada J., Savaram K., Travlou N.A., Li W., Li Q., Delgado-Sánchez C., Fierro V., Celzard A., He H., Bandosz T.J. (2017). Combined Effect of Porosity and Surface Chemistry on the Electrochemical Reduction of Oxygen on Cellular Vitreous Carbon Foam Catalyst. ACS Catal..

[36] Bandosz T.J. (2022). Revealing the impact of small pores on oxygen reduction on carbon electrocatalysts: A journey through recent findings. Carbon.

[37] Zhang D., Mitchell E., Lu X., Chu D., Shang L., Zhang T., Amal R., Han Z. (2023). Metal-free carbon-based catalysts design for oxygen reduction reaction towards hydrogen peroxide: From 3D to 0D. Mater. Today.

[38] Kislenko V.A., Pavlov S.V., Nikitina V.A., Kislenko S.A. (2024). Revision of the oxygen reduction reaction on N-doped graphenes by grand-canonical DFT. Phys. Chem. Chem. Phys..

[39] Wang N., Ma S., Zhang R., Wang L., Wang Y., Yang L., Li J., Guan F., Duan J., Hou B. (2023). Regulating N Species in N-Doped Carbon Electro-Catalysts for High-Efficiency Synthesis of Hydrogen Peroxide in Simulated Seawater. Adv. Sci..

[40] Li B., Sun X., Su D. (2015). Calibration of the basic strength of the nitrogen groups on the nanostructured carbon materials. Phys. Chem. Chem. Phys..

[41] Tan Z., Qin X., Cao P., Chen S., Yu H., Su Y., Quan X. (2023). Enhanced electrochemical-activation of H_2_O_2_ to produce •OH by regulating the adsorption of H_2_O_2_ on nitrogen-doped porous carbon for organic pollutants removal. J. Hazard. Mater..

[42] Zhang T., Zuo S. (2024). Nitrogen-doped metal-free granular activated carbons as economical and easily separable catalysts for peroxymonosulfate and hydrogen peroxide activation to degrade bisphenol A. Environ. Sci. Pollut. Res..

[43] Niu Y.-Q., Liu J.-H., Aymonier C., Fermani S., Kralj D., Falini G., Zhou C.-H. (2022). Calcium carbonate: Controlled synthesis, surface functionalization, and nanostructured materials. Chem. Soc. Rev..

[44] Takacs L. (2013). The historical development of mechanochemistry. Chem. Soc. Rev..

[45] James S.L., Adams C.J., Bolm C., Braga D., Collier P., Friščić T., Grepioni F., Harris K.D.M., Hyett G., Jones W. (2012). Mechanochemistry: Opportunities for new and cleaner synthesis. Chem. Soc. Rev..

[46] Liu X., Li Y., Zeng L., Li X., Chen N., Bai S., He H., Wang Q., Zhang C. (2022). A Review on Mechanochemistry: Approaching Advanced Energy Materials with Greener Force. Adv. Mater..

[47] Morawa Eblagon K., Rey-Raap N., Figueiredo J.L., Pereira M.F.R. (2021). Relationships between texture, surface chemistry and performance of N-doped carbon xerogels in the oxygen reduction reaction. Appl. Surf. Sci..

[48] Ribeiro R.S., Silva A.M.T., Figueiredo J.L., Faria J.L., Gomes H.T. (2013). The influence of structure and surface chemistry of carbon materials on the decomposition of hydrogen peroxide. Carbon.

[49] Vega E., Valdés H. (2018). New evidence of the effect of the chemical structure of activated carbon on the activity to promote radical generation in an advanced oxidation process using hydrogen peroxide. Microporous Mesoporous Mater..

