# Supplementary material for: Stability of Nitrogen-Doped Activated Carbon as an Electrocatalyst for the Oxygen Reduction Reaction in Various Storage Media
Source: Molecules. 2024 Jul 30;29(15):3611. doi: 10.3390/molecules29153611 (PMC11314166; doi:10.3390/molecules29153611)
Supplement: Supplementary file 1 [file molecules-29-03611-s001.zip › molecules-3126727-supplementary.pdf]

# Supporting Information

## Stability of nitrogen-doped activated carbon as an electrocatalyst for the oxygen reduction reaction in various storage media

Tao Zhang [1, 2], Songlin Zuo [1, 2,*]

[1] *College of Chemical Engineering, Nanjing Forestry University, International Innovation Center for Forest Chemicals and Materials, Nanjing, 210037, China*

[2] *Jiangsu Co-Innovation Center of Efficient Processing and Utilization of Forest Resources, Nanjing Forestry University, Nanjing, 210037, China*

[*] *Corresponding author. Tel: +86 25 85428840. E-mail address: zslnl@njfu.edu.cn*

**Text S1** Determination of BPA concentration

The concentration of BPA was analyzed by the high-performance liquid chromatography (HPLC, 1100, Agilent, USA) equipped with an ultraviolet detector (λ = 273 nm) and an Agilent Zorbax SB-$C_{18}$ chromatographic column (250 mm × 4.6 mm, 5 μm). The mobile phase was a mixture of acetonitrile and ultrapure water (60:40, v/v) and the flow rate was 0.8 mL/min. The column temperature was 30°C and the sample injection volume was 20 μL. The standard curve for detecting BPA was shown in this Figure

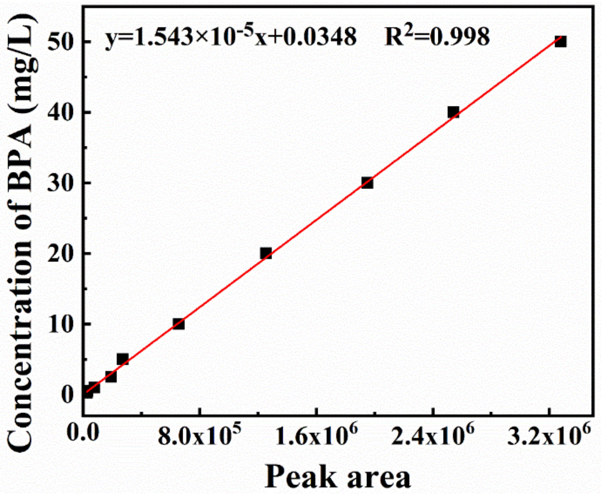


Standard curve of BPA (Concentration of BPA: 0.25~50 mg/L)

**Text S2** The determination of concentration of the basic sites of the carbons

To measure the basic active sites, 0.15 g of each carbon sample was added to a 100 mL conical flask containing a cover that contained 25 mL HCl solution (0.02 M). Then, the flask was placed in a shaking bath under stirring (200 rpm) for 48 h at 25°C. After filtration, 10 mL of the filtrate was pipetted out and the filtrate was back titrated with NaOH solution (0.02 M). Phenolphthalein (0.5 wt. %) was employed as the indicator for both titrations. The initial content of the acid active sites was calculated by the difference between the amount of initial HCl solution and the amount of HCl determined by back titration, and then dividing this value by the mass of carbon.

**Text S3** The determination of $pH_{pzc}$ of the samples

For each sample, fifteen conical flasks with covers were filled with 20 mL of 0.01 M NaCl solution. Then, 0.2 g of sample was added to each flask. Each flask was added with varying amounts of 0.1 M NaOH and 0.1 M HCl to ensure that the initial pH value of the solution was between 2 and 10. The final pH of the solution was measured with a pH meter after 48 h of shaking at 25°C. Hence, $pH_{pzc}$ is obtained from the point where the curve of final pH vs. initial pH crosses the line $pH_{initial} = pH_{final}$.

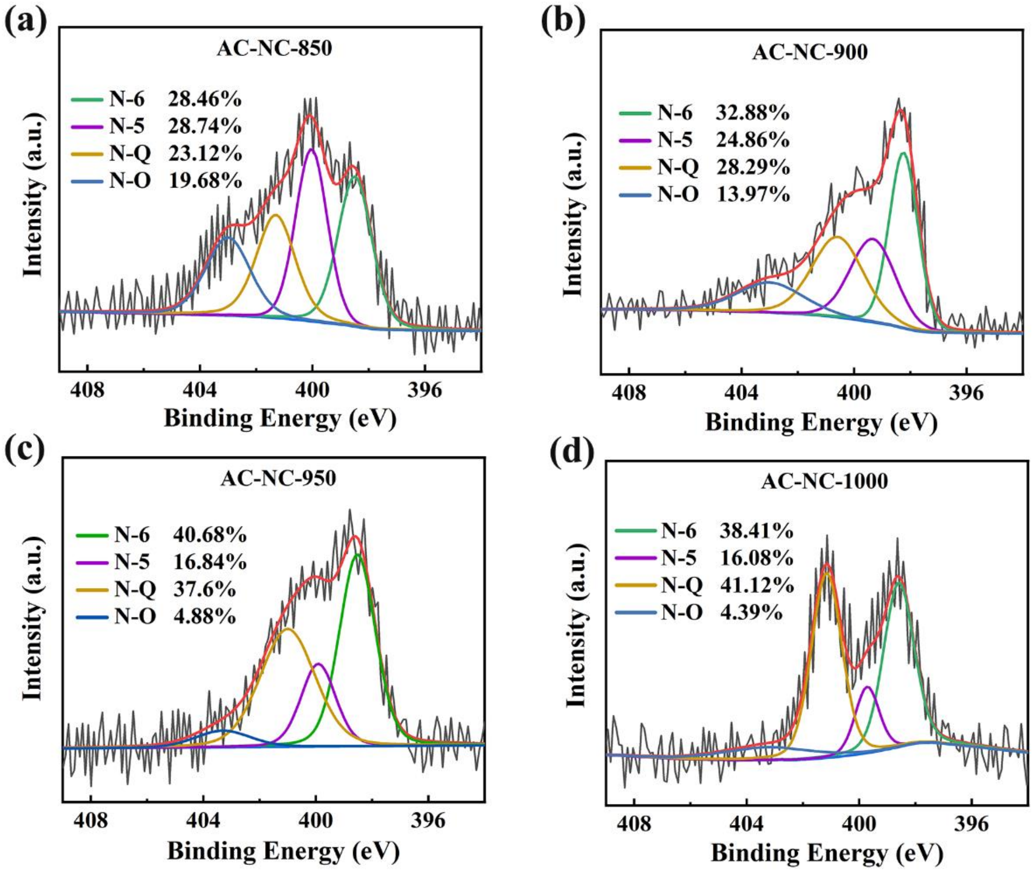


**Figure S1** (a) XPS survey spectrum and (b) high resolution N 1s of N-doped activated carbons.

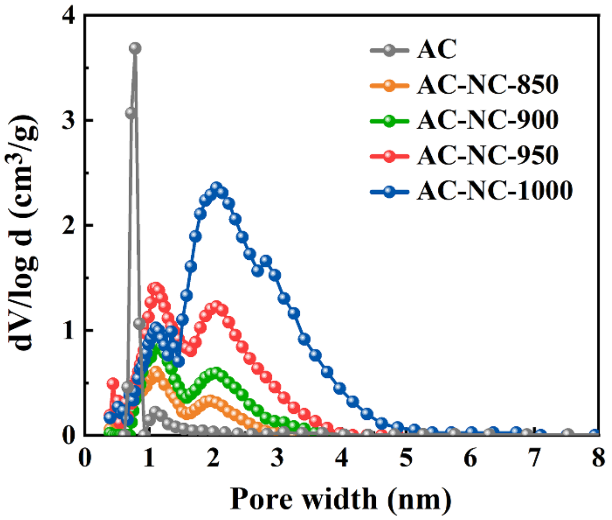


**Figure S2** Pore size distributions of the activated carbons.

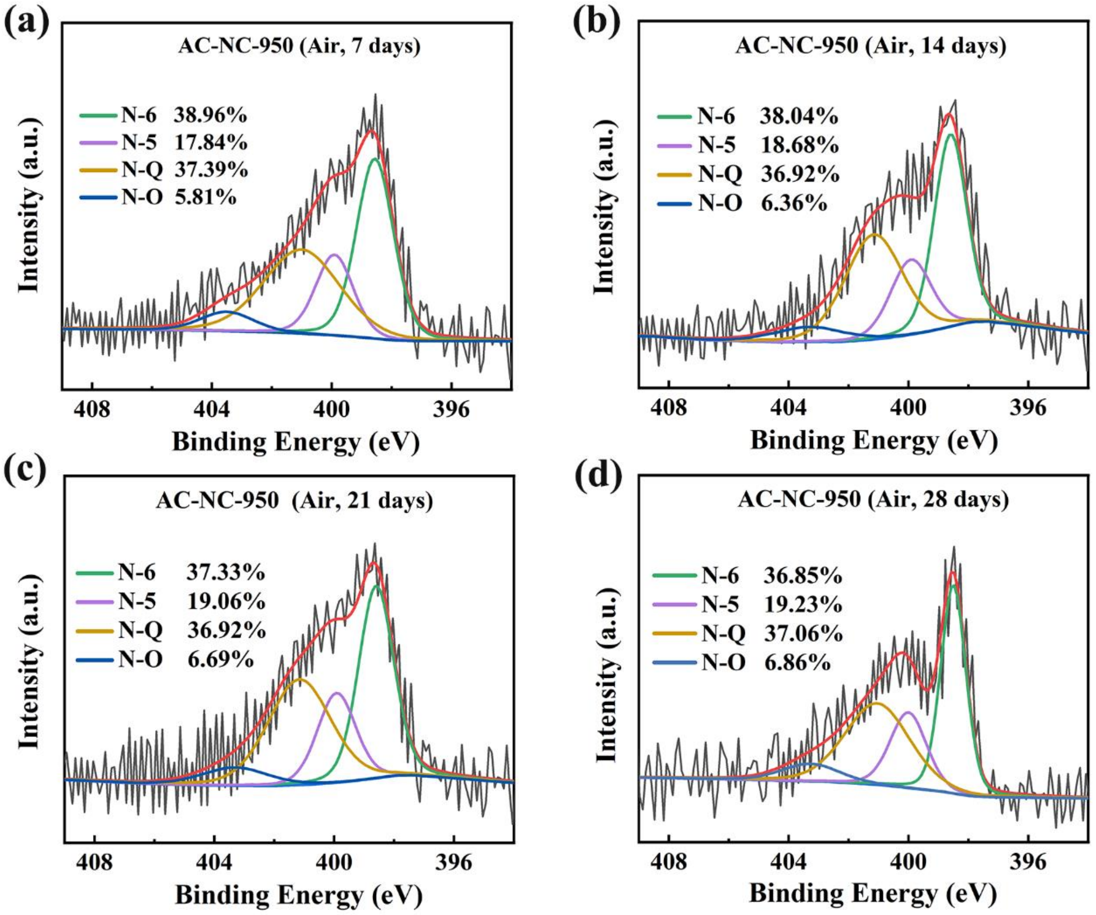


**Figure S3** High-resolution N 1s of XPS spectra of AC-NC-950 stored under air atmosphere for varying durations.

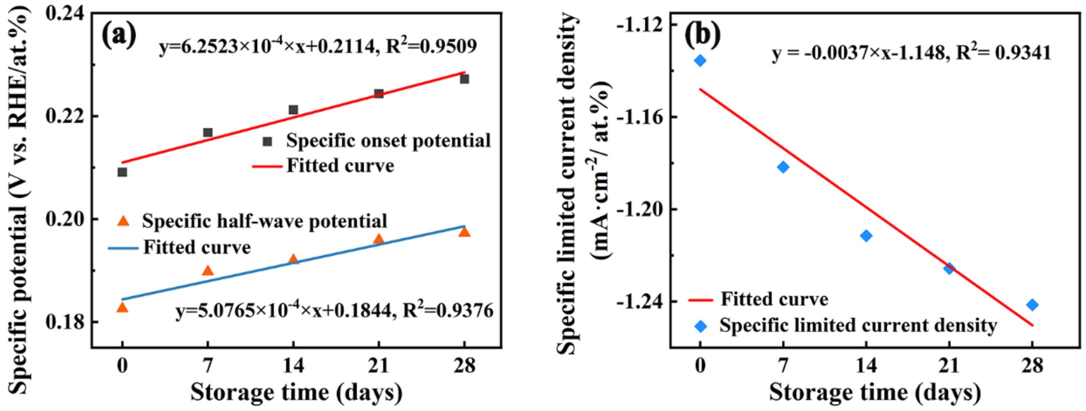


* The specific potential value equals the $E_{onset}$ or $E_{1/2}$ of the AC-NC-950 catalyzed ORR, which has been stored in air for a certain period, divided by the total absolute contents of N-6 and N-Q in the AC-NC-950 at that specific time.

* The specific limited current density value is determined by the $J_L$ of the AC-NC-950 catalyzed ORR, which has been stored under air environment for a certain period, divided by the total absolute contents of N-6 and N-Q in the AC-NC-950 at that specific time.

FigureS4 The evolution of the specific electrocatalytic activity of AC-NC-950 stored under air atmosphere.

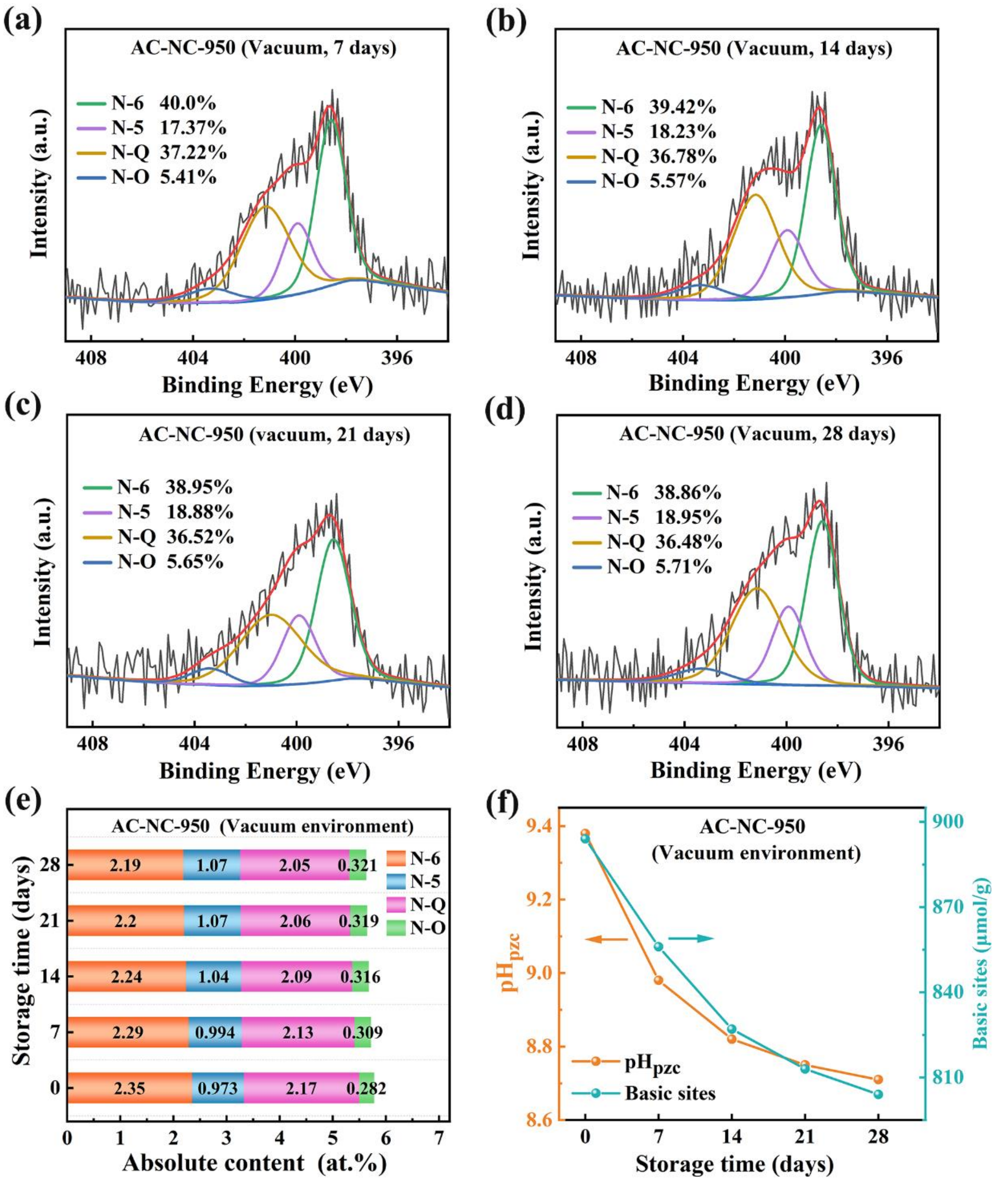


**Figure S5** (a-d) High-resolution XPS spectra of N 1s of the AC-NC-950 stored under vacuum atmosphere for varying durations. (e) The absolute content of each nitrogen-containing component in AC-NC-950 changes over time when stored in an air atmosphere. (f) The alkaline properties of AC-NC-950 following storage in vacuum environment for varying durations.

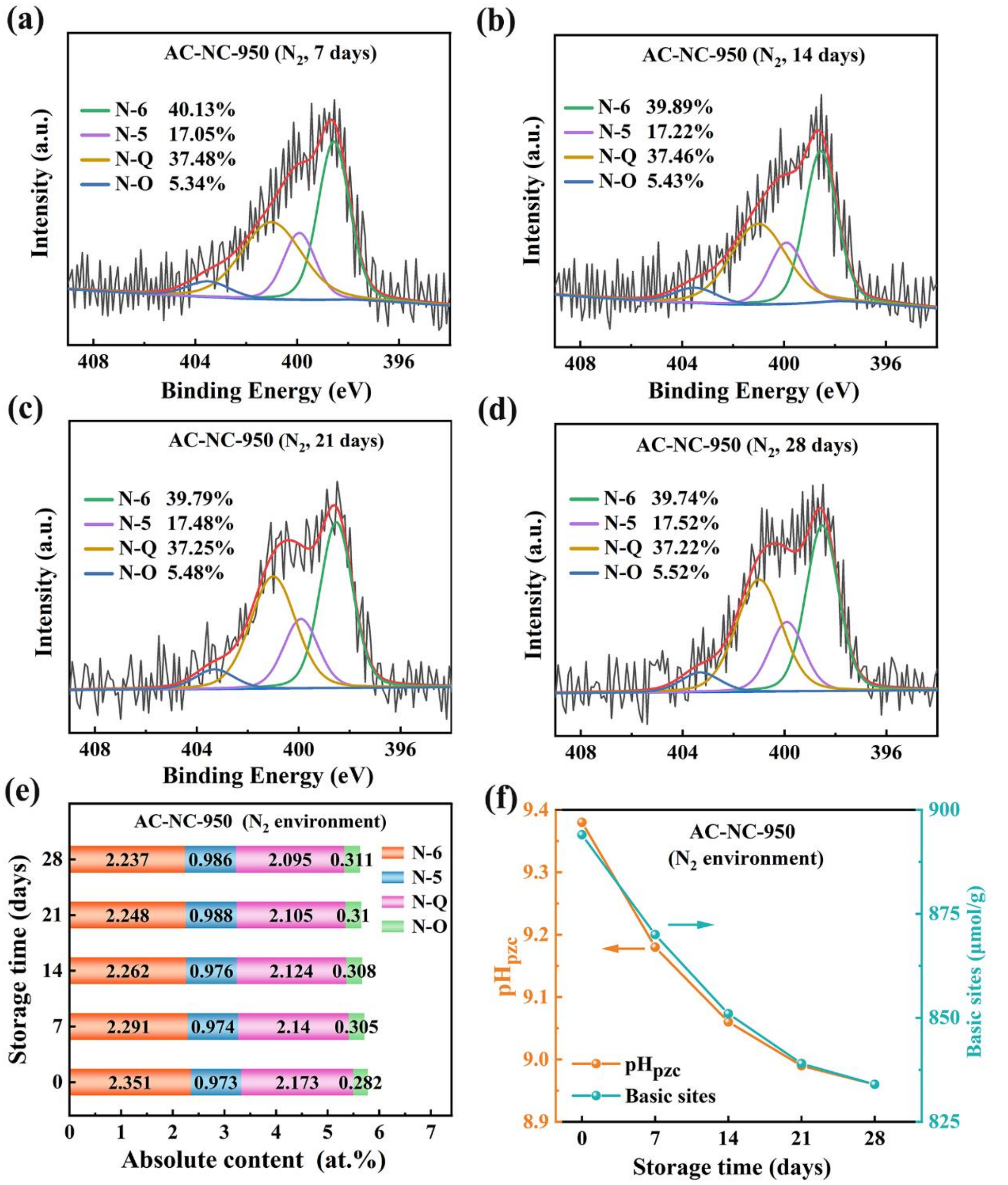


**Figure S6** (a-d) High-resolution XPS spectra of N 1s of the AC-NC-950 stored under a $N_2$ atmosphere for varying durations. (e) The absolute nitrogen content of AC-NC-950 stored in a $N_2$ environment changes over times. (f) The alkaline properties of AC-NC-950 following storage in a $N_2$ environment for varying durations.

**Table S1**

Textural and surface chemistry of AC and AC-NC-T.

| Sample | $S_{BET}$ ($m^2/g$) | $V_t$ ($cm^3/g$) | $V_{mic}$ ($cm^3/g$) | $V_{mes}$ ($cm^3/g$) | $V_{mes}/V_t$ (%) |
|---|---|---|---|---|---|
| AC | 986 | 0.426 | 0.379 | 0.047 | 11.0 |
| AC-NC-850 | 1121 | 0.441 | 0.364 | 0.077 | 17.5 |
| AC-NC-900 | 1294 | 0.521 | 0.402 | 0.119 | 22.8 |
| AC-NC-950 | 1536 | 0.708 | 0.415 | 0.293 | 41.4 |
| AC-NC-1000 | 1762 | 1.021 | 0.433 | 0.588 | 57.6 |

**Table S2a**

The elemental content changes of AC-NC-950 after being stored in different media (air, vacuum, and $N_2$) for different periods of time, as measured by elemental analysis.

| AC-NC-950 | | Element content by elemental analysis / wt.% | | | | | | | | | | | | | | | |
|---|---|---|---|---|---|---|---|---|---|---|---|---|---|---|---|---|---|
| Storage media | Storage time (days) | C[1] | C[2] | C* | SD (%) | H[1] | H[2] | H* | SD (%) | O[1] | O[2] | O* | SD (%) | N[1] | N[2] | N* | SD (%) |
| Newly prepared | 0 | 91.88 | 91.78 | 91.83 | 7.07 | 0.55 | 0.67 | 0.61 | 4.24 | 2.49 | 2.55 | 2.52 | 4.24 | 5.08 | 5 | 5.04 | 5.65 |
| Air | 7 | 91.62 | 91.72 | 91.65 | 4.24 | 0.52 | 0.64 | 0.58 | 4.24 | 2.99 | 2.85 | 2.93 | 8.48 | 4.87 | 4.78 | 4.84 | 4.24 |
| | 14 | 91.40 | 91.52 | 91.44 | 5.65 | 0.64 | 0.64 | 0.64 | 2.75 | 3.27 | 3.17 | 3.22 | 7.07 | 4.69 | 4.71 | 4.7 | 1.41 |
| | 21 | 91.44 | 91.48 | 91.46 | 2.82 | 0.71 | 0.53 | 0.62 | 6.36 | 3.27 | 3.31 | 3.29 | 2.82 | 4.58 | 4.68 | 4.63 | 7.07 |
| | 28 | 91.46 | 91.57 | 91.5 | 5.65 | 0.58 | 0.60 | 0.59 | 0.71 | 3.33 | 3.31 | 3.32 | 1.41 | 4.63 | 4.52 | 4.59 | 5.65 |
| Vacuum | 7 | 91.82 | 91.73 | 91.79 | 4.24 | 0.56 | 0.64 | 0.60 | 2.82 | 2.64 | 2.72 | 2.68 | 5.65 | 4.98 | 4.88 | 4.93 | 7.07 |
| | 14 | 91.79 | 91.71 | 91.75 | 5.65 | 0.56 | 0.70 | 0.63 | 4.94 | 2.73 | 2.75 | 2.74 | 1.41 | 4.92 | 4.82 | 4.88 | 5.65 |
| | 21 | 91.73 | 91.69 | 91.71 | 2.82 | 0.66 | 0.70 | 0.68 | 1.41 | 2.74 | 2.79 | 2.77 | 2.82 | 4.86 | 4.78 | 4.84 | 2.82 |
| | 28 | 91.78 | 91.78 | 91.81 | 4.24 | 0.61 | 0.55 | 0.58 | 2.12 | 2.75 | 2.82 | 2.79 | 4.24 | 4.85 | 4.77 | 4.82 | 4.24 |
| $N_2$ | 7 | 91.67 | 91.75 | 91.71 | 5.65 | 0.68 | 0.62 | 0.65 | 2.12 | 2.60 | 2.68 | 2.64 | 5.65 | 5.05 | 4.95 | 5 | 7.07 |
| | 14 | 91.62 | 91.7 | 91.65 | 4.24 | 0.75 | 0.63 | 0.69 | 4.24 | 2.65 | 2.75 | 2.7 | 7.07 | 4.98 | 4.94 | 4.96 | 2.82 |
| | 21 | 91.73 | 91.68 | 91.74 | 4.24 | 0.63 | 0.57 | 0.6 | 2.12 | 2.70 | 2.76 | 2.73 | 4.24 | 4.96 | 4.9 | 4.93 | 4.24 |
| | 28 | 91.63 | 91.72 | 91.68 | 7.07 | 0.70 | 0.56 | 0.63 | 4.95 | 2.74 | 2.82 | 2.78 | 5.65 | 4.93 | 4.89 | 4.91 | 2.82 |

[1]: the first elemental analysis result of sample AC-NC-950

[2]: the second elemental analysis result of sample AC-NC-950

*: the average of the results from two elemental analysis tests.

SD: stand for “standard deviation”

**Table S2b**

The elemental content changes of AC-NC-950 after being stored in different media (air, vacuum, and $N_2$) for different periods of time, as measured by XPS.

| AC-NC-950 | | Element content by XPS (at. %) | | | | | | | | | | | |
|---|---|---|---|---|---|---|---|---|---|---|---|---|---|
| Storage media | Storage time (days) | C[1] | C[2] | C* | SD (%) | O[1] | O[2] | O* | SD (%) | N[1] | N[2] | N* | SD (%) |
| Newly prepared | 0 | 90.87 | 90.88 | 90.875 | 0.71 | 3.35 | 3.42 | 3.385 | 4.95 | 5.78 | 5.7 | 5.74 | 5.65 |
| Air | 7 | 90.84 | 90.86 | 90.85 | 1.41 | 3.64 | 3.69 | 3.665 | 3.53 | 5.52 | 5.45 | 5.485 | 4.94 |
| | 14 | 90.85 | 90.95 | 90.9 | 7.07 | 3.82 | 3.81 | 3.815 | 0.71 | 5.33 | 5.24 | 5.285 | 6.36 |
| | 21 | 90.87 | 90.91 | 90.89 | 2.82 | 3.92 | 3.98 | 3.95 | 4.24 | 5.21 | 5.11 | 5.16 | 7.07 |
| | 28 | 90.87 | 90.92 | 90.895 | 3.53 | 3.98 | 4.03 | 4.005 | 3.53 | 5.15 | 5.05 | 5.1 | 7.07 |
| Vacuum | 7 | 90.84 | 90.87 | 90.855 | 2.12 | 3.50 | 3.53 | 3.515 | 2.12 | 5.66 | 5.6 | 5.63 | 4.24 |
| | 14 | 90.83 | 90.95 | 90.89 | 8.48 | 3.57 | 3.53 | 3.55 | 2.82 | 5.60 | 5.52 | 5.56 | 5.65 |
| | 21 | 90.84 | 90.88 | 90.86 | 2.82 | 3.61 | 3.62 | 3.615 | 0.71 | 5.55 | 5.5 | 5.525 | 3.53 |
| | 28 | 90.84 | 90.85 | 90.845 | 0.71 | 3.64 | 3.69 | 3.665 | 3.53 | 5.52 | 5.46 | 5.49 | 4.24 |
| $N_2$ | 7 | 90.87 | 90.84 | 90.855 | 2.12 | 3.42 | 3.49 | 3.455 | 4.94 | 5.71 | 5.67 | 5.69 | 2.82 |
| | 14 | 90.83 | 90.87 | 90.85 | 2.82 | 3.50 | 3.51 | 3.505 | 0.71 | 5.67 | 5.62 | 5.645 | 3.53 |
| | 21 | 90.82 | 90.84 | 90.83 | 1.41 | 3.53 | 3.58 | 3.555 | 3.53 | 5.65 | 5.58 | 5.615 | 4.94 |
| | 28 | 90.82 | 90.83 | 90.825 | 0.71 | 3.55 | 3.6 | 3.575 | 3.53 | 5.63 | 5.57 | 5.6 | 4.24 |

[1]: the first elemental analysis result of sample AC-NC-950

[2]: the second elemental analysis result of sample AC-NC-950

*: the average of the results from two elemental analysis tests.

SD: stand for “standard deviation”

**Table S3**

The LSV curve parameters obtained from AC-NC-950 after being stored in a vacuum and a $N_2$ atmosphere for various durations.

| Samples (AC-NC-950) | | $E_{onset}$ | $E_{1/2}$ | $J_L$ |
|---|---|---|---|---|
| Storage media | Storage time (days) | (V vs. RHE) | (V vs. RHE) | (mA $cm^{-2}$) |
| Newly prepared | 0 | 0.946 | 0.827 | -5.122 |
| Vacuum | 7 | 0.938 | 0.817 | -5.013 |
| | 14 | 0.928 | 0.809 | -4.996 |
| | 21 | 0.920 | 0.804 | -4.988 |
| | 28 | 0.917 | 0.801 | -4.975 |
| $N_2$ | 7 | 0.940 | 0.819 | -5.032 |
| | 14 | 0.931 | 0.811 | -4.997 |
| | 21 | 0.924 | 0.807 | -4.995 |
| | 28 | 0.921 | 0.805 | -4.992 |
